# Arteriovenous Malformation of the Superficial Femoral Artery: A Case Report

**DOI:** 10.7759/cureus.65975

**Published:** 2024-08-01

**Authors:** Jasleen Dua, Renuka S Jadhav, Mridu Bahal, Shailaja Mane, Aryan Gupta

**Affiliations:** 1 Pediatrics, Dr. D. Y. Patil Medical College, Hospital and Research Centre, Dr. D. Y. Patil Vidyapeeth (Deemed to be University), Pune, IND

**Keywords:** pediatric arteriovenous malformations, ct angiography, congenital vascular malformation, peripheral arteriovenous malformation, superficial femoral artery

## Abstract

Peripheral arteriovenous malformations (AVMs) are rare vascular anomalies characterized by abnormal connections between arteries and veins that bypass the capillary system. This case report details a three-year-old female child who presented with an enlarging swelling on her knee's medial side. AVM was diagnosed using computed tomography (CT) angiography and surgically excised. The case highlights the importance of early detection and timely intervention of AVMs to prevent complications.

## Introduction

Peripheral arteriovenous malformations (AVMs) in children, although rare, present significant diagnostic and therapeutic challenges due to their complex nature [1]. AVMs are abnormal connections between arteries and veins that bypass the capillary system, leading to direct high-flow shunts [2]. This abnormal blood flow can cause various complications depending on the size and location of the lesion [3]. In children, AVMs often manifest with symptoms such as pain, ulceration, bleeding, and even heart failure due to the increased cardiac output needed to manage the abnormal blood flow [4,5]. Diagnosing these lesions can be particularly challenging because their symptoms can mimic other more common conditions. Advanced imaging techniques such as magnetic resonance imaging (MRI), computed tomography (CT) scans, and angiography are typically required to accurately identify and assess the extent of AVMs [6].

Extracranial AVMs can occur throughout the body, but they are most frequently found in the head and neck region, followed by the lungs and extremities [7]. AVMs in the head and neck can lead to significant functional and aesthetic issues, given the complexity and density of structures in these areas. In the lungs, AVMs can cause significant respiratory complications and hypoxemia, while those in the extremities can result in severe limb pain, swelling, and ulceration [8]. The diverse presentation of AVMs, ranging from asymptomatic lesions to those causing significant morbidity, underscores the need for a high index of suspicion and thorough evaluation in pediatric patients presenting with unexplained symptoms.

## Case presentation

**Figure 1:**
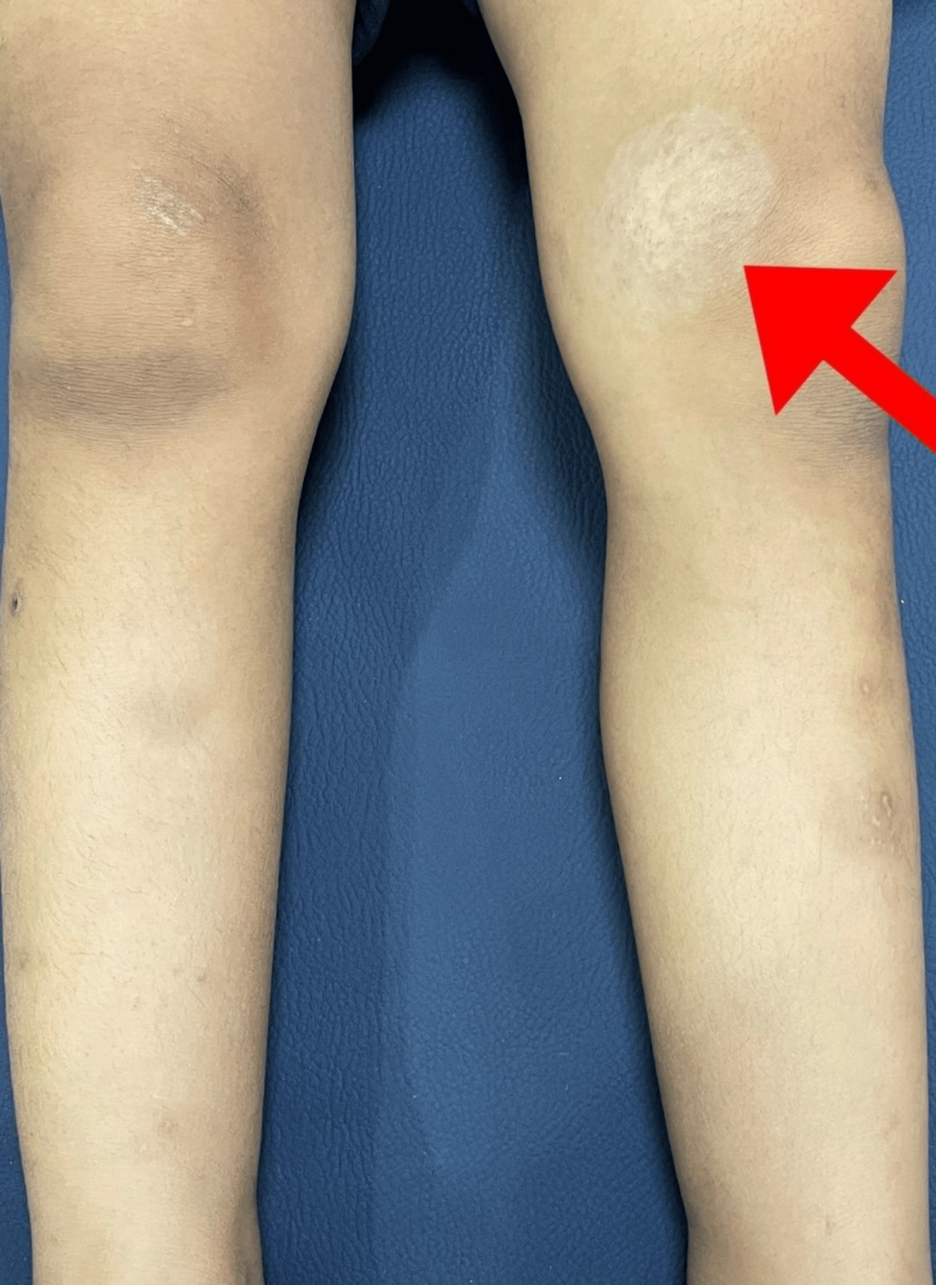
Clinical picture showing arteriovenous malformation Red arrow: Swelling present over the medial aspect of the knee 5×5 cm with multiple petechiae over it

A three-year-old girl, born third via normal vaginal delivery, presented with a progressively enlarging swelling on the medial side of her left knee since birth. Initially measuring 0.8×0.5 cm, the swelling had grown to 5×5 cm over time. There was no history of bleeding, pain, trauma, or ulceration at the site, nor were there any similar lesions elsewhere on her body. Clinical examination revealed a localized swelling on the left knee's medial side, measuring 5×5 cm with a circular shape, regular borders, and soft consistency. The swelling was non-tender and pulsatile with a palpable thrill. The affected area was warmer than the surrounding skin and exhibited multiple petechiae. The mass was fixed to underlying structures and not freely mobile (Figure 1).

Based on the history and physical examination, an AVM was strongly suspected. There was no family history of similar lesions.

**Figure 2:**
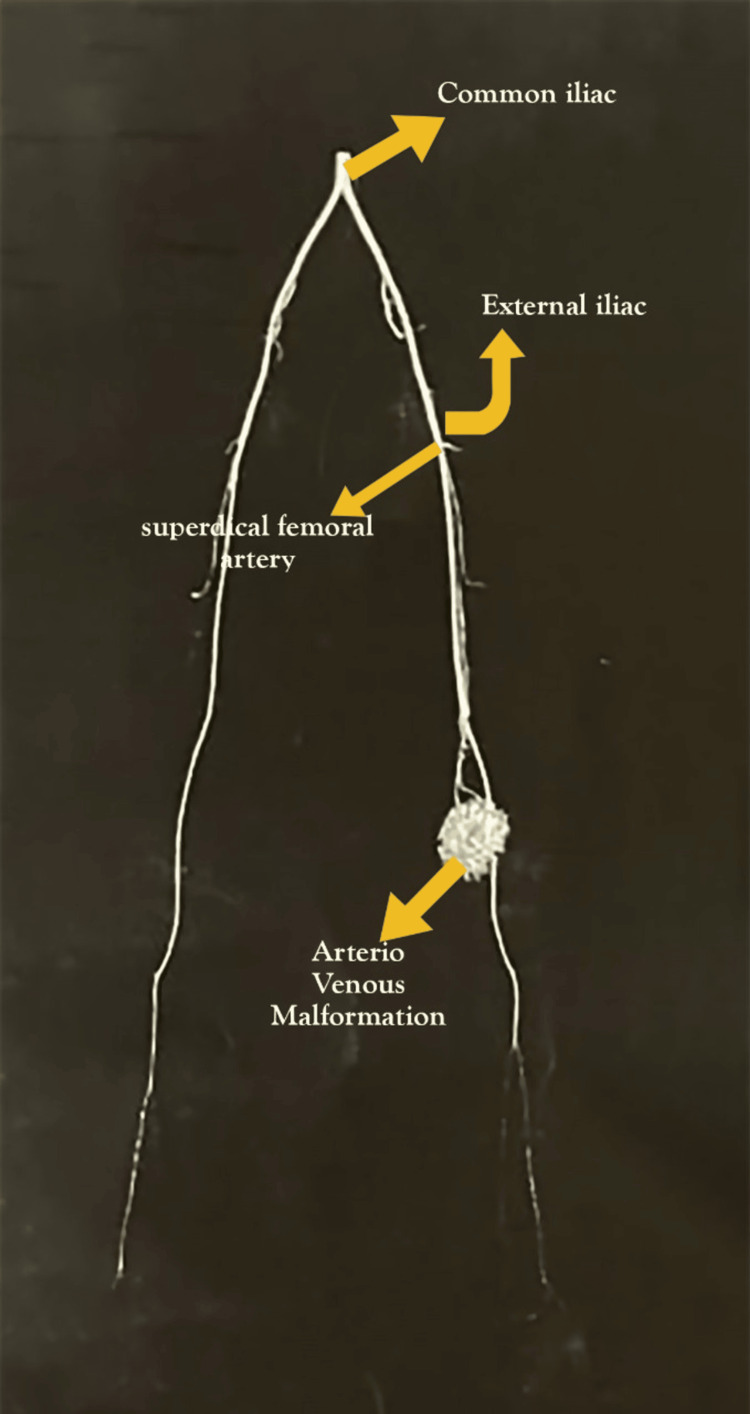
CT lower limb angiography CT bilateral lower limb angiography revealed a well-defined, avidly enhancing subcutaneous lesion measuring 4.5×1.3×3.9 cm on the medial aspect of the left knee. Enlarged muscular branches of the superficial femoral artery were supplying the lesion, with the largest branch arising approximately 7.7 cm proximal to the knee. Multiple venous channels were observed draining into the superficial femoral vein above the knee, with early enhancement noted in the arterial phase. CT: computed tomography

Diagnostic imaging was conducted, and CT bilateral lower limb angiography revealed a well-defined, avidly enhancing subcutaneous lesion measuring 4.5×1.3×3.9 cm on the medial aspect of the left knee. Enlarged muscular branches of the superficial femoral artery were supplying the lesion, with the largest branch arising approximately 7.7 cm proximal to the knee. Multiple venous channels were observed draining into the superficial femoral vein above the knee, with early enhancement noted in the arterial phase (Figure 2).

These findings confirmed the diagnosis of AVM. Additionally, the patient had normal results on 2D echocardiography and ultrasonography of the abdomen and pelvis, ruling out any other associated anomalies.

The treatment plan included surgical excision and primary repair of the AVM. Postoperative care focused on pain management, infection prevention through antibiotics, and regular follow-up to monitor for recurrence or complications. The patient recovered well with no immediate postoperative complications. At a six-month follow-up, she was asymptomatic, had normal limb function, and showed no signs of recurrence. Long-term follow-up was scheduled to monitor for any new vascular anomalies or late recurrences, ensuring ongoing management of her condition.

## Discussion

AVMs are uncommon vascular anomalies arising from developmental defects. These lesions result from aberrant connections between arteries and veins that bypass the capillary system, leading to high-flow shunts characterized by thick-walled arteries and arterialized veins [1,2]. AVMs account for 10-20% of all vascular malformations, with an estimated incidence of one in 100,000 individuals [3,5]. They occur without a clear gender or ethnic predilection, and their exact cause remains unknown. AVMs can appear in isolation or as part of genetic syndromes, such as PTEN (Phosphatase and TENsin) hamartoma syndromes, Parkes Weber syndrome, hereditary hemorrhagic telangiectasia, and capillary malformation AVM syndrome [9].

AVMs are congenital anomalies that grow in proportion to the individual and do not regress spontaneously. Their growth follows a predictable pattern with four distinct stages: quiescent, growing, symptomatic, and decompensating [10]. The Schobinger clinical staging system categorizes AVMs as follows: Stage I (quiescent) is characterized by asymptomatic presentation with a pink-blue skin blush and warmth, detectable shunting via Doppler. Stage II (expansion) involves lesion enlargement with tortuous veins, pulsation, and the presence of a bruit or palpable thrill. Stage III (destruction) includes pain, dystrophic skin changes, ulceration, skin necrosis, bleeding, distal ischemia, and steal phenomena. Stage IV (decompensation) is marked by the aforementioned changes along with high-output cardiac failure [10,11].

Their tendency to progress often results in clinical manifestations during the second or third decade of life. The clinical presentations can vary widely, from minimal symptoms to severely disabling conditions, impacting psychological and emotional well-being. The presentation depends on the AVM's location, duration, and size. AVMs in areas with visible cutaneous manifestations, such as the face and scalp, tend to present earlier than those in the extremities. Although these lesions are usually not painful, there have been reports of painful cases [12].

The high-flow nature of AVMs contributes to significant morbidity and mortality risks [3]. These lesions can cause vascular steal syndromes, where the AVM diverts blood flow from normal tissues, potentially leading to ischemia. In severe cases, they may induce high-output heart failure due to the increased cardiac workload needed to sustain the abnormal blood flow. Long-standing cutaneous AVMs are prone to frequent bleeding due to venous hypertension and stasis changes, which can result in persistent ulceration and chronic pain [13]. Given these potential complications, early detection and intervention are critical to preventing adverse outcomes.

A case study by Ghareeb et al. highlights the consequences of delayed intervention in AVMs. The study discusses an eight-year-old girl who presented with a necrotic sacral gluteal mass accompanied by pain and bleeding. MRI revealed a large, non-capsulated superficial soft tissue mass. After embolization to reduce blood flow, the patient underwent successful excisional surgery [7]. This contrasts with the patient in the present report, who presented early without bleeding, allowing for timely surgical excision and prevention of complications. This underscores the importance of early clinical assessment and the critical role of imaging in the diagnosis and treatment planning of AVMs, emphasizing the need for early intervention to avoid severe complications and improve patient outcomes.

In contrast to previous studies, the patient in this report presented early without bleeding, and early surgical excision prevented complications. The use of CT angiography was instrumental in confirming the diagnosis of an AVM underscoring the importance of thorough clinical assessment and the role of imaging in diagnosis and treatment planning.

## Conclusions

This case report underscores the importance of early detection and intervention in managing peripheral AVMs in children. The timely surgical excision of an enlarging knee swelling in a three-year-old girl prevented significant complications. Advanced imaging, specifically CT angiography, was crucial for accurate diagnosis and treatment planning. Early recognition and appropriate management of AVMs can lead to excellent outcomes, reducing the risk of severe complications such as bleeding, ulceration, and heart failure. This case highlights the necessity for healthcare providers to be vigilant in identifying AVMs and utilizing advanced diagnostic tools for optimal patient care.
